# The lantibiotic gallidermin acts bactericidal against *Staphylococcus epidermidis* and *Staphylococcus aureus* and antagonizes the bacteria‐induced proinflammatory responses in dermal fibroblasts

**DOI:** 10.1002/mbo3.606

**Published:** 2018-03-13

**Authors:** Torbjörn Bengtsson, Johanna Lönn, Hazem Khalaf, Eleonor Palm

**Affiliations:** ^1^ Department of Medical Sciences Örebro University Örebro Sweden; ^2^ Department of Oral Biology Institute of Odontology Malmö University Malmö Sweden; ^3^ PEAS Research Institute Linköping Sweden

**Keywords:** antimicrobial resistance, bacteriocin, cytokines, fibroblasts, gallidermin, Streptococcus

## Abstract

Antimicrobial resistance needs to be tackled from new angles, and antimicrobial peptides could be future candidates for combating bacterial infections. This study aims to investigate in vitro the bactericidal effects of the lantibiotic gallidermin on *Staphylococcus epidermidis* and *Staphylococcus aureus*, possible cytotoxic effects and its impact on host‐microbe interactions. Minimal inhibitory concentration (MIC) and minimal bactericidal concentration (MBC) of gallidermin were determined, and cytotoxicity and proinflammatory effects of gallidermin on fibroblasts, red blood cells (RBCs) and in whole blood were investigated. Both MIC and MBC for all four tested strains of *S. epidermidis* was 6.25 μg/ml. Both MIC and MBC for methicillin‐sensitive *S. aureus* was 12.5 μg/ml and for methicillin‐resistant *S. aureus* (MRSA) 1.56 μg/ml. Gallidermin displayed no cytotoxic effects on fibroblasts, only a high dose of gallidermin induced low levels of CXCL8 and interleukin‐6. Gallidermin hemolyzed less than 1% of human RBCs, and did not induce reactive oxygen species production or cell aggregation in whole blood. In cell culture, gallidermin inhibited the cytotoxic effects of the bacteria and totally suppressed the bacteria‐induced release of CXCL8 and interleukin‐6 from fibroblasts. We demonstrate that gallidermin, expressing low cell cytotoxicity, is a promising candidate for treating bacterial infections caused by *S. epidermidis* and *S. aureus*, especially MRSA.

## 1 INTRODUCTION

The fast evolution of antimicrobial resistance makes the world once more facing the same challenges in human and veterinary medicine as it did before Alexander Fleming's discovery of penicillin 1928 (Brown & Wright, 2016; Fleming, 2001). Infections and infectious diseases as well as medical treatments, such as surgical procedures, organ transplantations, prosthetic implantations, chemotherapy and so forth, are yet again potential risks for adverse effects with increased morbidity and mortality due to antimicrobial resistance (Hughes, 2011). Antimicrobial resistance also brings a high economic burden for the public health care system, including higher medical expenses and longer hospital stays. If no actions are taken, the fast development of antibiotic resistance is estimated, from the current approximated 700,000 deaths each year, to by year 2050 globally causing 10 millions of deaths annually, which is even more than cancer, making it the leading cause of death, at a cost of US$100 trillion (Piddock, 2016; Rossolini, Arena, Pecile, & Pollini, 2014). Therefore, there is an urgent need for developing new antimicrobial strategies and agents. Currently, there are no antibiotic in clinical use to which resistance has not been developed (Asaduzzaman & Sonomoto, 2009).

Antimicrobial peptides are a promising field for the discovery of novel antibiotics. These peptides are virtually found in all living organisms, including bacteria, fungi, plants, invertebrates, and vertebrates. Most of these antimicrobial compounds are small cationic and amphiphatic peptides that are an important component of the organisms innate immune defense (Ageitos, Sanchez‐Perez, Calo‐Mata, & Villa, 2017). Bacteria constitute a rich source of antimicrobial peptides, referred to as bacteriocins. Bacteriocins are ribosomally synthesized peptides that often target closely related bacterial species, and are used to overcome each other in the competition of space and nutrients (Cavera, Arthur, Kashtanov, & Chikindas, 2015). There are several groups of bacteriocins, classified according to their biochemical and genetic properties. One of these classes is lanthionine‐containing antibiotic peptides, simply abbreviated as lantibiotics. The group of lantibiotics comprises globular or elongated peptides that are characterized by containing the unusual amino acid lanthionine and/or 3‐methylanthionine (Dischinger, Basi Chipalu, & Bierbaum, 2014). Posttranslational modification involves formation of the unusual amino acids and renders the lantibiotic biologically active (Asaduzzaman & Sonomoto, 2009; Sahl & Bierbaum, 1998). Due to high‐quality genomic data provided by next generation sequencing technologies combined with the development of specific genome mining tools, the number of known lantibiotics, including knowledge of their biosynthetic pathways, increase each year (Sandiford, 2017). Lantibiotics are produced by many gram‐positive species, with a substantial amount derived from commensal staphylococci, but other species such as *Lactococcus*,* Bacillus*,* Streptomyces*,* Pediococcus* and *Streptococcus* also produce lantiobiotics (Gotz, Perconti, Popella, Werner, & Schlag, 2014). The antibacterial effects of lantibiotics generally depend on inhibition of cell wall biosynthesis by binding to the cell wall precursor lipid II, and/or pore formation with disruption of membrane integrity, mechanisms that for some lantibiotics are initiated by high bacterial affinities due to electrostatic forces of the cationic lantibiotic to the anionic bacterial cell envelope. Gram‐negative bacteria are in general not affected by lantibiotics due to its protective outer membrane (Dischinger et al., 2014).

Nisin that was discovered already in the 1920s is the most studied lantibiotic (Rogers, 1928). Nisin induces pores that span the cell membrane through interaction with lipid II. This pore consists of four lipid II and eight nisin molecules (Hasper, de Kruijff, & Breukink, 2004). Pore formation leads to an efflux of metabolites from the bacteria and disruption of vital ion gradients (Sahl & Brandis, 1982; Wiedemann et al., 2001). Since lipid II is a cell wall precursor, the binding of nisin to lipid II also inhibits transglycosylation by steric hindrance, resulting in the sequestration of lipid II and, hence, in its abduction from cell wall synthesis (Breukink et al., 2003; Brotz et al., 1998). The interaction primarily involves formation of five hydrogen bonds between the pyrophosphate moiety of lipid II and the amide backbone of the *N*‐terminal ring pair of nisin (Hsu et al., 2004). The lipid II binding motif of nisin is conserved among a number of lantibiotics such as epidermin, plantaricin C, and subtilin (Willey & van der Donk, 2007). Gallidermin, discovered and structurally characterized in the 1980s, belongs to the lantibiotics and is produced by *Staphylococcus gallinarum* (Kellner et al., 1988; Schnell et al., 1989). Gallidermin, sharing the same lipid II binding motif as nisin, is also able to inhibit cell wall biosynthesis. However, since gallidermin is, with its 22 amino acids, considerably shorter than nisin, the peptide is not able to span the membrane and, hence, its mode of action is primarily cell wall biosynthesis inhibition rather than pore formation (Bonelli, Schneider, Sahl, & Wiedemann, 2006; Willey & van der Donk, 2007). So far, gallidermin has shown promising bactericidal effects against gram‐positive species and was found to be more efficient than vancomycin against *Micrococcus luteus* (Maher & McClean, 2006). In addition, gallidermin is efficient against *Staphylococcus epidermidis*,* Staphylococcus aureus, Proponiumbacterium acnes*,* Peptostreptococcus anaerobicus* as well as *Streptococcus pyogenes* (Kellner et al., 1988; Saising et al., 2012).

This study aims to evaluate the bactericidal effects of gallidermin on *S. epidermidis* and *S. aureus* in an in vitro model of infection as well as the capacity of gallidermin to induce cytotoxic and proinflammatory responses in dermal fibroblasts, red blood cells (RBCs), and whole blood. We demonstrate that gallidermin is a promising candidate for treating bacterial infections caused by *S. epidermidis* and *S. aureus*, especially methicillin‐resistant *S. aureus* (MRSA).

## 2 MATERIALS AND METHODS

### 2.1 Bacterial strains and preparation

Four strains of *S. epidermidis* were used in this study; biofilm‐negative ATCC 12228, biofilm‐positive RP62A, strain N15 isolated from a healthy, nonhospitalized person, and strain 117 isolated from a prosthetic hip infection. Methicillin‐sensitive *S. aureus* (MSSA) ATCC 29213 and methicillin‐resistant MRSA CCUG 35601 were also included in the study. All strains were kindly provided by Professor Bo Söderqvist and PhD Bengt Hellmark at the Department of Laboratory Medicine, Clinical Microbiology, Örebro University Hospital, Sweden. Cultures were maintained on blood agar plates. For each strain, respectively, one bacterial colony was transferred from the blood agar plate to 5 ml of Luria‐Bertani (LB) broth (Difco Laboratories, Detroit, MI, USA). Bacterial strains were grown aerobically over night at 37°C on a shaker at 200 rpm. The bacteria were harvested by centrifugation at 9,300*g* for 10 min, washed in phosphate‐buffered saline (PBS, Invitrogen Ltd., Paisley, UK), and resuspended in LB broth before use.

### 2.2 Minimal inhibitory concentration and minimal bactericidal concentration

Minimal inhibitory concentration (MIC) was determined by the broth microdilution method according to the Clinical and Laboratory Standards Institute (Methods for Dilution Antimicrobial Susceptibility Tests for Bacteria That Grow Aerobically; Approved Standard: Tenth Edition. CSLI document M07‐A10. 2015). Briefly, gallidermin, with a purity of 94.3%, (Santa Cruz Biotechnology, Inc., Dallas, TX, USA) was serial diluted in PBS into a 96‐well microtiter plate (TC plate, 96 well, suspension, F, Sarstedt, Inc, Newton, NC, USA), at a volume of 100 μl. The bacterial suspension was prepared as described above and bacterial density was adjusted so that 100 μl of LB broth corresponded to a bacterial inoculum of 10^5^ colony‐forming units (CFU) of bacteria. After adding 100 μl of bacterial suspension to each well, resulting in a final volume of 200 μl, the final concentrations of gallidermin ranged from 0.19 to 100 μg/ml (0.09 to 46.18 μmol/L). The plate was incubated aerobically at 37°C for 24 hr on a shaker at 200 rpm. The bacterial growth was assessed by reading optical density (OD) in a microplate reader at 620 nm (Multiskan Ascent, Thermo Labsystem, Stockholm, Sweden). MIC was determined as the lowest concentration of gallidermin that inhibited the bacterial growth.

For the determination of MBC, 10 μl of bacterial suspension from each well that lacked visual turbidity, was plated on Mueller‐Hinton plates (BD Corporation, Franklin Lakes, New Jersey, USA). The plates were incubated at 37°C for 24 hr. Formed colonies were counted and MBC was determined as the lowest bactericidal concentration that resulted in at least 99.9% reduction in bacterial growth compared to the initial 10^5^ CFU.

### 2.3 Isolation and culture conditions of human primary dermal fibroblasts

Primary fibroblasts were isolated by explanting pieces of dermis obtained from elective chest surgery from a young adult donor at the Department of surgery, Örebro University hospital, Sweden. The skin was removed by standard surgical procedure. Approval from the local Ethical Committee at Örebro County Council, Sweden (no. 2003/0101) and informed, written consent from the patient were obtained. The surgical procedure and all experiments were performed in accordance with the Swedish national board of health and welfare guidelines and the ethical guidelines of the Helsinki‐declaration. Fibroblasts were propagated from dermal preparations by the explant technique, whereas small pieces (half‐millimeter) of dermis adhered to culture plastic. After a few minutes, Dulbecco's modified Eagle medium (DMEM) with 10% fetal bovine serum (FBS) and 1 mg/ml gentamicin were added (all from Invitrogen Ltd.). Fibroblasts were cultured at 37°C in 5% CO_2_ and passaged, (TrypLE^™^ Express Enzyme, ThermoFisher Scientific, Waltham, MA, USA), after reaching confluence. After the initial cell passages, gentamicin was removed. Fibroblasts were used at passage 6–13 without antibiotics.

### 2.4 Cell viability and cell cytotoxicity

Primary human dermal fibroblasts in 10% FBS DMEM were seeded in 24‐well plates (TC plate, 24 well, standard, F, Sarstedt, Inc) at a density of 50,000 cells/well. After overnight attachment at 37°C in 5% CO_2_, medium was replaced by serum‐free DMEM. After 24 hr of serum‐starvation, media was exchanged to 1% FBS DMEM 1 hr prior to stimulation. For cell viability and cytotoxicity, gallidermin was added: 25, 50, 100, 200, and 400 μg/ml, respectively (corresponding concentrations: 11.54, 23.09, 46.18, 92.35, and 184.71 μmol/L). After 24 hr, microscopy images were taken at 200x magnification (Olympus CKX41, Olympus Optical, Solna, Sweden). Cell culture supernatants were aliquoted and stored at −80°C for further analysis. For cell viability, neutral red (Sigma‐Aldrich, Darmstadt, Germany) was diluted in PBS (4 mg/ml) whereupon the solution was mixed 1:200 in DMEM without FBS and incubated at 37°C over night. Cells were washed twice in PBS and the neutral red solution was centrifuged at 600*g* for 10 min and sterile‐filtered before 500 μl was added to each well. Cells were incubated for 2 hr, washed with PBS and destained with 1 ml of neutral red destain solution (50% ethanol 96%, 49% MQ water, 1% glacial acetic acid). The plate was agitated for 10 min and the extracted neutral red OD was read at 540 nm. Viable cells were calculated as percent of the control according to the formula (abs sample/abs control) × 100 = viable cells in % of the control.

### 2.5 Reactive oxygen species and cell aggregation in whole blood

Reactive oxygen species (ROS) production and cell aggregation was measured simultaneously in whole blood using a lumiaggregometer (Chrono‐Log Model 700, Chrono‐Log Corporation, Havertown, PA, USA). The production of ROS was registered as luminol‐amplified chemiluminescence and cell aggregation as increase in impedance (Ω) between two platinum electrodes. Blood samples were collected according to the Swedish national board of health and welfare guidelines and the ethical guidelines of the Helsinki declaration. All blood samples were immediately anonymized and no information of blood donors was taken. Venous blood from four healthy volunteers was drawn into heparin tubes and used within 20 min before the onset of the experiment. Informed consent was obtained from all subjects. To each cuvette, 100 μmol/L 5‐amino‐2,3‐dihydro‐1,4‐phthalazinedione (Luminol), 8 U/ml horse‐radish peroxidase (HRP; both from Sigma‐Aldrich Co., St. Louis, MO, USA) and blood, diluted 1:1 with calcium‐free Krebs–Ringers glucose buffer (KRG; 120 mmol/L NaCl, 4.9 mmol/L KCl, 1.2 mmol/L MgCl_2_ × 7H_2_O, 1.7 mmol/L KH_2_PO_4_, 8.3 mmol/L Na_2_HPO_4_ × 2H_2_O and 10 mmol/L glucose in water), were added. After 10 min of preincubation at 37°C, 50 μg/ml (23.09 μmol/L) or 200 μg/ml (92.35 μmol/L) of gallidermin was added. 0.5 μmol/L *N*‐Formylmethionyl‐leucyl‐phenylalanine (fMLP, Sigma‐Aldrich Co.) was used as a positive control and KRG was added to the negative control. Samples were under constant stirring using siliconized magnetic bars rotating at 800 rpm during the preincubation and the following measurements lasting for 15 min. The integral value of the chemiluminescence response and the level of aggregation was measured 15 min after addition of gallidermin, and compared to negative and positive control. The instrument was calibrated before each experiment.

### 2.6 Determination of lactate dehydrogenase, CXCL8, and interleukin‐6

To evaluate cytotoxic and immune responses of fibroblasts, lactate dehydrogenase (LDH), CXCL8, and IL‐6 were analyzed in cell culture supernatants. CytoSelect LDH Cytotoxicity Assay Kit (Nordic BioSite, Täby, Sweden) was performed according to the manufacturer's protocol. LDH values were calculated as the relative change from the control, sample/control = LDH release, setting the control to 1. The protein levels of CXCL8 and IL‐6, secreted by fibroblasts, were determined by human interleukin‐8 enzyme‐linked immunosorbent assay (ELISA) Standard Set and Human interleukin‐6 ELISA MAX Standard Set, respectively (both from Biolegend, San Diego, CA, USA), according to the manufacturer's recommendations.

### 2.7 Hemolytic assay

The release of hemoglobin from RBCs was measured and correlated to the fraction of cells that underwent osmotic hemolysis. Blood samples were collected according to the Swedish national board of health and welfare guidelines as well as the ethical guidelines of the Helsinki declaration. Informed consent was obtained from all subjects, no information of blood donors was taken and blood samples were immediately anonymized. Venous blood from three healthy persons was drawn into heparin tubes and centrifuged at 600*g* for 5 min. Plasma was removed and the remaining RBC fraction was washed with PBS and thereafter diluted with PBS to a 30% RBC fraction. 100 μl of the RBC suspension was mixed with 100 μl of gallidermin diluted in PBS, resulting in final gallidermin concentrations ranging from 25 to 400 μg/ml (11.54 to 184.71 μmol/L) in a 96‐well microtiter plate (TC plate, 96 well, suspension, F, Sarstedt, Inc). Triton‐X (1%) treated RBCs served as positive control and nontreated RBCs as negative control. The samples were incubated at 37°C for 1 hr. Hemoglobin concentration was determined by reading OD at 540 nm (Multiskan Ascent, Thermo Labsystem). Hemolysis was calculated as (Absorbance Sample/Absorbance positive control) × 100 = % hemolysis. The positive control was defined as 100% hemolysis.

### 2.8 The effects of gallidermin on bacteria‐induced activation of fibroblasts

For bacterial stimulation of primary human dermal fibroblasts, the cells were prepared as described above for the cytotoxicity test. Briefly, cells in 10% FBS DMEM were seeded at a density of 50,000 cells/well in 24‐well plates (TC plate, 24 well, standard, F, Sarstedt, Inc). After overnight attachment, fibroblasts were serum‐starved for 24 hr before medium was swapped to 1% FBS DMEM 1 hr prior to stimulation. Viable *S. epidermidis* or *S. aureus* were added at a multiplicity of infection (MOI) of 1 and 10, respectively, with or without gallidermin. The concentration of gallidermin used was 6.25 μg/ml (2.89 μmol/L) for *S. epidermidis* and 12.5 μg/ml (5.77 μmol/L) for *S. aureus*. The chosen gallidermin concentrations correlated to the highest determined MIC value for *S. epidermidis* and *S. aureus*, respectively. The bacterial cell suspension was incubated for 24 hr at 37°C in 5% CO_2_, upon which microscopy images were obtained at 100x magnification (Olympus CKX41, Olympus Optical). Culture supernatants were aliquoted and stored at −80°C for further analysis. Concentrations of CXCL8 and IL‐6 were determined as described above.

### 2.9 Statistical analysis

Data of individually performed experiments are presented as mean with standard error of the mean (SEM) indicated. MBCs are represented by median values. Statistical comparisons between individual groups were performed by one‐way analysis of variance (ANOVA) with Bonferroni post hoc test using GraphPad Prism5 (GraphPad software, La Jolla, CA, USA). Differences were considered to be statistically significant when *p* values were *p < .05, **p < .01, ***p < .001. All experiments were repeated at least three times.

## 3 RESULTS

### 3.1 Gallidermin inhibits bacterial growth and act bactericidal on *S. epidermidis* and *S. aureus*

**Figure 1:**
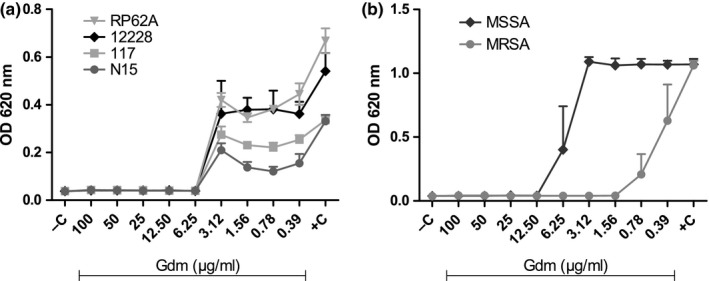
Gallidermin inhibits the growth of *Staphylococcus epidermidis* and *Staphylococcus aureus*. Minimal inhibitory concentration (MIC) after treatment with gallidermin (Gdm) were determined for four strains of *S. epidermidis*: biofilm‐negative ATCC 12228, biofilm‐positive RP62A, strain N15 isolated from a healthy person, and strain 117 isolated from a prosthetic hip infection (a). MIC and MBC were also determined for two strains of *S. aureus;* methicillin‐sensitive MSSA ATCC 29213 and methicillin‐resistant MRSA CCUG 35601 (b). Gallidermin was diluted in a 96‐well microtiter plate with concentrations ranging from 0.19 to 100 μg/ml. Bacteria (10^5^ CFU/100 μl) were added and the suspensions was incubated at 37°C for 24 hr. Optical density (OD) was read at 620 nm after 24 hr. MIC correlates to the lowest concentration of gallidermin that inhibits bacterial growth. Negative control without bacteria (−C) and positive control with bacteria but without gallidermin (+C) are also shown. Data are presented as mean values with SEM indicated. *n *= 3

The minimal inhibitory concentration (MIC) is the lowest concentration of an antimicrobial compound that inhibits bacterial growth and minimal bactericidal concentration (MBC) is the lowest bactericidal concentration. MIC of gallidermin for the four included strains of *S. epidermidis* was 6.25 μg/ml (2.89 μmol/L) (Figure 1a). MBC also gave 6.25 μg/ml for all tested *S. epidermidis* strains (Table 1). For methicillin‐sensitive *S. aureus* (MSSA) MIC (Figure 1b) as well as MBC of gallidermin was 12.5 μg/ml (5.77 μmol/L). Gallidermin was very efficient against methicillin‐resistant (MRSA) with MIC and MBC 1.56 μg/ml (0.72 μmol/L). MIC and MBC results are summarized in Table 1.

**Table 1:** Gallidermin MIC for *Staphylococcus epidermidis* and *Staphylococcus aureus*

	MIC and MBC (μg/ml)	MIC and MBC (μmol/L)
*12228*	6.25	2.89
*117*	6.25	2.89
*RP62A*	6.25	2.89
*N15*	6.25	2.89
*MSSA*	12.5	5.77
*MRSA*	1.56	0.72

Minimal inhibitory concentration (MIC) and minimal bactericidal concentration (MBC) after treatment with gallidermin (μg/ml) were determined for four strains of *S. epidermidis* (12228, 117, RP62A, and N15) and two strains of *S. aureus* (MSSA and MRSA). Gallidermin was diluted in a 96‐well microtiter plate with concentrations ranging from 100 to 0.19 μg/ml. Bacteria (10^5^ CFU/100 μl) were added and incubated at 37°C for 24 hr. MIC correlates to the lowest concentration that inhibits bacterial growth. Optical density was read at 620 nm. For MBC, 10 μl of bacterial suspension was streaked on blood agar plates, incubated for 24 hr and the lowest bactericidal concentration was determined. MIC was the same as the MBC for each individual strain. Corresponding concentrations in micromole is also given. Median values are shown. *n *= 3.

### 3.2 Gallidermin shows low cytotoxicity and induces a low immune response in fibroblasts

**Figure 2:**
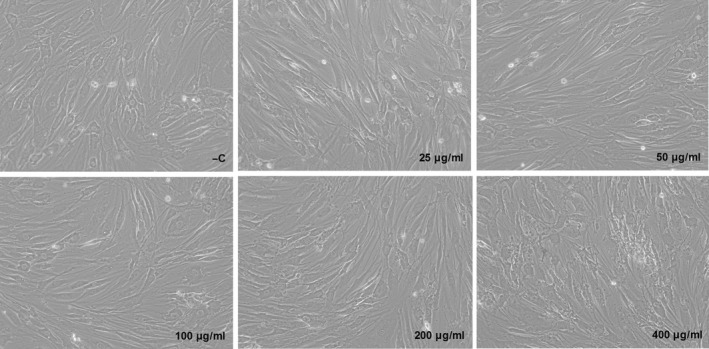
Morphological effects of gallidermin on dermal fibroblasts. Human primary dermal fibroblasts (50,000 cells/well) were treated with various concentrations of gallidermin (25–400 μg/ml) for 24 hr, followed by images taken at a magnification of 200x. Fibroblasts without gallidermin served as negative control (−C). Representative images are shown from one experiment. *n *= 3

**Figure 3:**
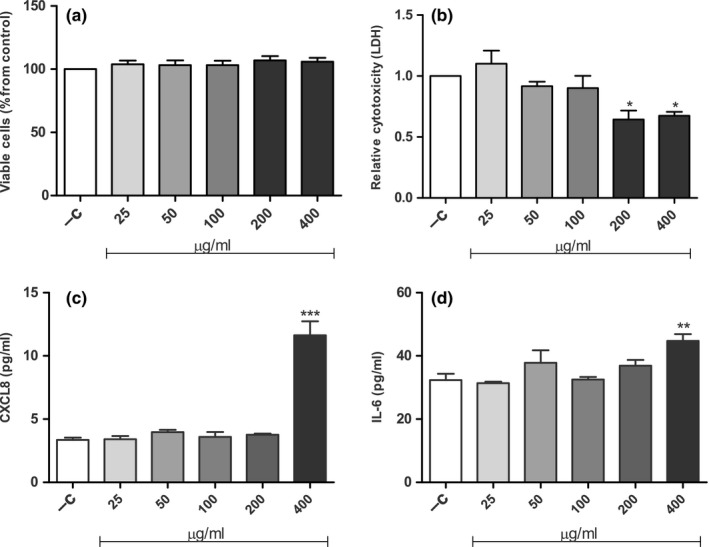
The effects of gallidermin on viability, cytotoxicity and immune responses of dermal fibroblasts. Human primary dermal fibroblasts (50,000 cells/well) were untreated (−C) or treated with various concentrations of gallidermin (25–400 μg/ml) for 24 hr. Viability was determined as uptake of neutral red by viable cells (a). As a measure of cell cytotoxicity, the absorbance values of LDH release were measured and analyzed as the relative change from the negative control (nontreated fibroblasts) set to one (b). Secretion of the proinflammatory mediator CXCL8 was determined with ELISA and showed a significant increase when fibroblasts were treated with 400 μg/ml gallidermin, whereas lower concentrations had no effects on CXCL8 (c). IL‐6 displayed a similar pattern, i.e. only 400 μg/ml gallidermin significantly elevated IL‐6 (d). Data are presented as mean values with SEM indicated. Statistically significant differences were determined by using one‐way ANOVA with Bonferroni *post hoc* test (*p < 0.05, **p < 0.01, ***p < 0.001). *n *= 3

Primary human dermal fibroblasts were exposed to various doses of gallidermin for 24 hr to evaluate possible cellular effects. No major alterations of cell density or morphology could be seen at 25–100 μg/ml (11.54–46.18 μmol/L) of gallidermin, compared to the negative control. However, the two highest tested concentrations, 200 and 400 μg/ml (92.35 and 187.71 μmol/L), induced minor alterations in fibroblast morphology although the cell number visually appeared to be equal to the control (Figure 2). Viability determined as the uptake of neutral red by viable, metabolically active cells, showed that none of the tested doses of gallidermin affected the viability of the fibroblasts (Figure 3a). In addition, the extent of cell cytotoxicity was evaluated by determining the release of LDH, since an increase in cell death rate is accompanied by an extracellular accumulation of LDH. Surprisingly, the two highest concentrations, 200 and 400 μg/ml gallidermin, significantly reduced LDH levels compared to the control, whereas at lower doses LDH release was similar to the control (Figure 3b). Besides determining cytotoxic effects of gallidermin, we also evaluated the capacity of gallidermin to induce an inflammatory response in fibroblasts. Analysis of fibroblast release of the proinflammatory mediator CXCL8 showed that only the highest tested dose of gallidermin (400 μg/ml) significantly increased the amount of this chemokine from 3 to 12 pg/ml (Figure 3c). Similar outcome was found for interleukin‐6 (IL‐6), where 400 μg/ml gallidermin caused a significant increase from 32 to 44 pg/ml (Figure 3d).

### 3.3 Gallidermin causes no hemolysis, cell aggregation, or reactive oxygen species production in whole blood

**Figure 4:**
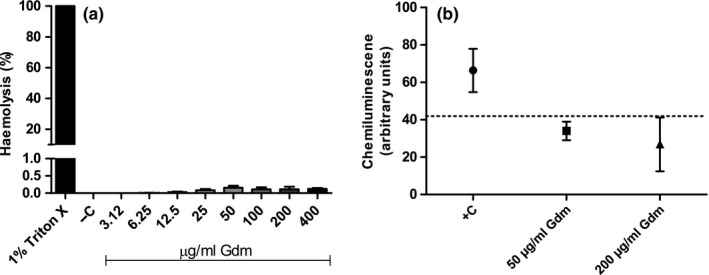
Hemolysis and reactive oxygen species production in whole blood treated with gallidermin. Human red blood cells (RBCs), diluted to a 30% RBCs fraction in PBS, were treated with gallidermin (Gdm) for 1 hr at 37°C, and optical density was read at 540 nm (a). 1% Triton X‐treated RBCs served as a positive control (+C), giving 100% hemolysis. All tested gallidermin concentrations caused less than 1% of hemolysis. Gallidermin (50 or 200 μg/ml) was added to whole blood and reactive oxygen species (ROS) production was measured as luminol‐amplified chemiluminescence (b). Whole blood without stimulation served as a negative control and is indicated by the dotted line. Whole blood stimulated with fMLP (0.5 μmol/L) served as a positive control (+C). Data are presented as mean values with SEM indicated. Statistically significant differences compared to the negative control were determined by using one‐way ANOVA with Bonferroni post hoc test. *n *= 3–4

The resistance of RBCs membranes against different agents and drugs can be used as a method of measuring toxicity. Hence, the capacity of gallidermin to interfere with human RBCs membrane integrity was evaluated. We found that all tested concentrations of gallidermin only had marginal effects resulting in hemolysis lower than 1%, and with no differences compared to the untreated, negative control (Figure 4a).

The ability of gallidermin to interfere with blood cells was also evaluated by measuring ROS production and cell aggregation in whole blood through luminol‐amplified chemiluminescence and impedance, respectively. As a positive control, the chemotactic peptide fMLP (0.5 μmol/L) was used which is known to induce both cell aggregation and ROS in whole blood (Follin, Johansson, & Dahlgren, 1991; Losche, Redlich, Krause, Heptinstall, & Spangenberg, 1998). None of the tested concentrations of gallidermin induced ROS production in whole blood (Figure 4b). Gallidermin displayed ROS at levels that were comparable to the negative control. In the same way, none of the two concentrations of gallidermin were able to induce cell aggregation and did not differ from the control. Mean value of negative control was 8.67 Ω, for 50 μg/ml (23.09 μmol/L) gallidermin 7.25 Ω and for 200 μg/ml (92.35 μmol/L) gallidermin 5.22 Ω (results not shown). Number of experiment (*n*) was 4.

### 3.4 Gallidermin counteracts the cellular effects induced by *S. epidermidis* and *S. aureus*

**Figure 5:**
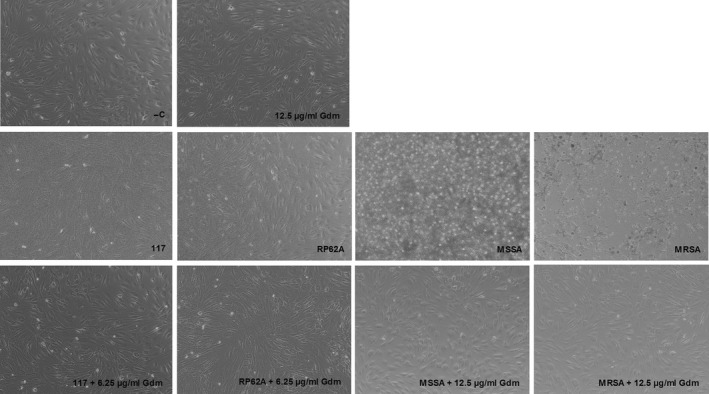
Morphological effects of *Staphylococcus epidermidis*,* Staphylococcus aureus* and gallidermin on dermal fibroblasts. *S. epidermidis* 117, *S. epidermidis* RP62A, MSSA, or MRSA was incubated with human primary dermal fibroblasts (50,000 cells/well) at MOI:10, in the absence or presence of gallidermin (Gdm), with 6.25 μg/ml for *S. epidermidis* and 12.5 μg/ml for *S. aureus*. The cells were incubated for 24 hr at 37°C, whereupon microscopic images were captured with at a magnification of 100x. As a positive control, fibroblasts were stimulated with 6.25 μg/ml (not shown) or 12.5 μg/ml gallidermin and nontreated fibroblasts were used as a negative control (−C). Representative images are shown. *n *= 3

**Figure 6:**
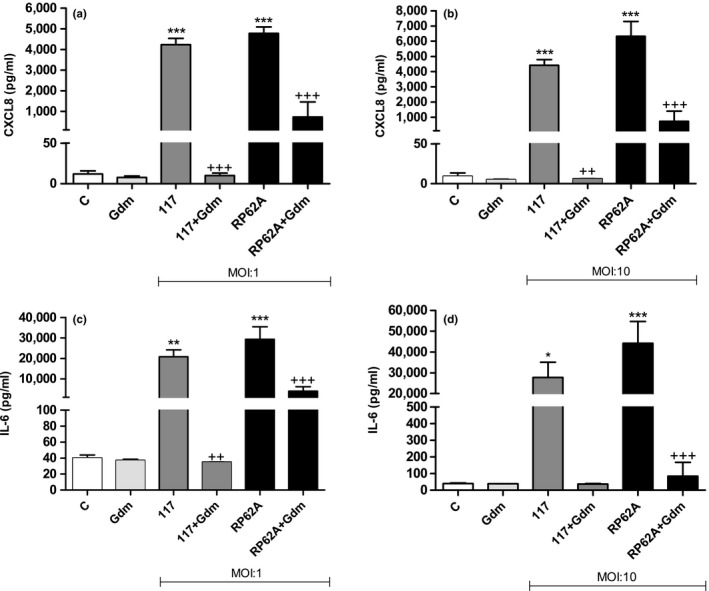
Gallidermin suppresses the inflammatory responses of *Staphylococcus epidermidis*‐stimulated fibroblasts. Human primary dermal fibroblasts (50,000 cells/well) were treated with *S. epidermidis* with or without 6.25 μg/ml gallidermin (Gdm) for 24 hr at 37°C. Cell culture supernatants were analyzed for CXCL8 and IL‐6 released from fibroblasts stimulated with the bacterial strain 117 or RP62A at MOI:1 and MOI:10. Nontreated fibroblasts were used as a negative control (‐C). Strain 117 and RP62A raised the CXLC8 level whereas gallidermin suppressed this induction at MOI:1 (a) as well as MOI:10 (b). IL‐6 was also elevated by 117 and RP62, but was counteracted when gallidermin was added at MOI:1 (c) and MOI:10 (d). Data are presented as mean values with SEM indicated. Statistically significant differences were determined by using one‐way ANOVA with Bonferroni post hoc test (*p < 0.05, **/++*p* < 0.01, ***/+++*p* < 0.001). *Statistically significant differences compared to ‐C. +Statistically significant differences compared to corresponding bacterial strain without gallidermin. *n *= 3

To elucidate the influence of gallidermin in an in vitro cell system, dermal fibroblasts were treated with *S. epidermidis* strain 117 and RP62A at MOI:1 and MOI:10, respectively. Microscopic images show prominent bacterial growth when the tested strains were added to cell cultures at MOI:10. However, in the presence of gallidermin, bacterial growth of all tested strains were restricted (Figure 5). Similar results were obtained at MOI:1 (not shown). Both *S. epidermidis* 117 and RP62A (MOI:1 and MOI:10) markedly elevated the release of CXCL8 from fibroblasts (Figure 6). Gallidermin itself did not affect the level of CXCL8. Strain 117 and RP62A at MOI:1 released high amounts of IL‐6 from fibroblasts (Figure 6c). As with CXCL8, gallidermin significantly reduced bacteria‐induced secretion of IL‐6. The reduction was most prominent for strain 117. However, at MOI:10, the gallidermin‐mediated decrease in IL‐6 triggered by strain 117 was not statistically secured even though the difference was as prominent as for MOI:1 and for RP62A (Figure 6d). *S. epidermidis* RP62A markedly increased the release of IL‐6 from fibroblasts, an effect that was totally abolished by gallidermin.

**Figure 7:**
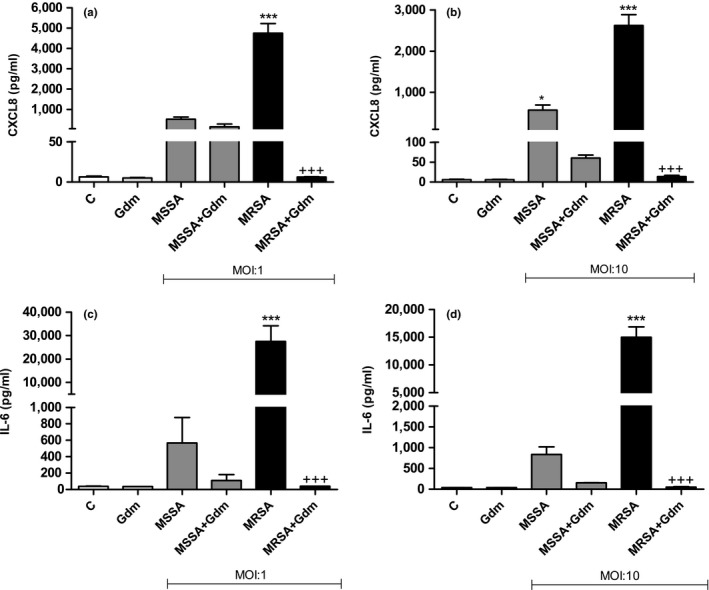
Gallidermin suppresses the inflammatory responses of *Staphylococcus aureus*‐stimulated fibroblasts. Human primary dermal fibroblasts (50,000 cells/well) were treated with MSSA and MRSA with or without 12.5 μg/ml gallidermin (Gdm) for 24 hr at 37°C. Cell culture supernatants were analyzed for CXCL8 (a, b) and IL‐6 (c, d) released from fibroblasts stimulated with MSSA or MRSA at MOI:1 and MOI:10. Nontreated fibroblasts were used as a negative control (−C). MRSA induced CXCL8 secretion, whereas gallidermin inhibited this induction at MOI:1 (a). At MOI:10, both MSSA and MRSA resulted in high CXCL8 levels although gallidermin suppressed this CXCL8 accumulation from MRSA‐treated fibroblasts (b). The same pattern for MRSA was seen for IL‐6 (c, d). Data are presented as mean values with SEM indicated. Statistically significant differences were determined by using one‐way ANOVA with Bonferroni post hoc test (*p < 0.05, ***/+++*p* < 0.001). *Statistically significant differences compared to ‐C. +Statistically significant differences compared to corresponding bacterial strain without gallidermin. *n *= 3

The effects of gallidermin on *S. aureus* infection of fibroblasts were also studied. MRSA and MSSA at MOI:1 induced CXCL8 release from fibroblasts, but only MRSA caused statistically significant higher levels compared to the control (Figure 7a). MRSA‐induced CXCL8 was profoundly reduced by gallidermin, which was not the case for MSSA (Figure 7b). Surprisingly, MRSA at MOI:10 induced a lower release of CXCL8 from fibroblasts, approximately 2,600 pg/ml, in comparison to 4,800 pg/ml at MRSA MOI:1. MRSA induced at both MOI:1 and MOI:10 a substantial release of IL‐6 from fibroblasts, whereas MSSA (MOI:1 and MOI:10) caused a more modest elevation (Figure 7). Gallidermin totally antagonized MRSA‐stimulated generation of IL‐6 from fibroblasts to a level similar with the control. No significant differences regarding MSSA, with or without gallidermin were found. As seen with CXCL8, MRSA at MOI:10 induced a lower release of IL‐6 level from fibroblasts compared with MOI:1 (25,000 pg/ml and 18,000 pg/ml, respectively).

## 4 DISCUSSION

The use, and the extensive misuse, of antibiotics creates a strong evolutionary pressure for the emergence of resistant bacteria, throwing the world into a postantibiotic era where veterinary and human medical care may stand effortless in combating infections caused by multiresistant pathogens (Hughes, 2011). For instance, around 70% of the used antibiotics in the USA are utilized in agriculture as animal growth promotors (Sarmah, Meyer, & Boxall, 2006). Without effective antibiotics, surgery as well as cancer chemotherapy will be compromised (Hughes, 2011). Part from start to handle existing antibiotics with caution, the need for developing new antibiotics as well as new treatment strategies is of outermost importance. When antibiotics as efficient drugs are missing, also the foundation for a great part of today's modern health care will be lost.

The increasing resistance of pathogenic bacteria to conventional antibiotics makes antimicrobial peptides a promising alternative for the treatment of infections (Ageitos et al., 2017). In this study, we investigate the bactericidal and cell cytotoxic effects of the lantibiotic gallidermin. MIC and MBC of gallidermin were determined for four different strains of *S. epidermidis* (6.25 μg/ml). In comparison, MIC and MBC of gallidermin for MSSA was higher (12.5 μg/ml). MRSA on the other hand, showed a much higher susceptibility (1.56 μg/ml). The results demonstrate that gallidermin has a highly bactericidal effect against *S. epidermidis* and *S. aureus*. Kellner et al. (1988) demonstrated a MIC of 4 μg/ml gallidermin for both *S. aureus* and *S. epidermidis*, which was in accordance with another study that demonstrated similar susceptibilities to gallidermin for both *S. epidermidis* and *S. aureus* (4–8 μg/ml) (Saising et al., 2012). In comparison, MIC values of nisin against *S. aureus* strains were shown to be in the range of 2–32 μg/ml (Dosler & Gerceker, 2012), whereas another study demonstrated values of 2.5–10.1 μg/ml. Furthermore, nisin inhibited different strains of *S. epidermidis* in the range of 2.1–3.0 μg/ml (Mota‐Meira, LaPointe, Lacroix, & Lavoie, 2000), whereas another study reported MIC at 100 μg/ml (Broadbent, Chou, Gillies, & Kondo, 1989). Antimicrobial agents can be divided two classes, one class composed of mainly bacteriostatic agents and one class constituting the agents that are mainly bactericidal. For bactericidal agents, the MBC is usually the same as, and generally not more than fourfold greater than, the MIC (Levison & Levison, 2009). This suggests that gallidermin could be classified as a primarily bactericidal agent.

So far only a few studies suggest that bacteriocins may have proliferative and cell supporting effects besides the bactericidal properties. Both proliferation and migration of keratinocytes have been shown in a previous study to be promoted by the antimicrobial peptide plantaricin A from *Lactobacillus plantarum* (Pinto et al., 2011). In correlation, both published and unpublished data from our group demonstrate that plantaricin NC8 αβ, also produced by *L. plantarum*, induces growth factors in human gingival epithelial cells and stimulates cell proliferation (Bengtsson et al., 2017). An additional property of lantibiotics, which contributes to their promising potential as future therapeutic antimicrobial agents, is that lantibiotics usually display low cell cytotoxicity (Maher & McClean, 2006). We found that fibroblasts showed minor changes in morphology when exposed to high concentrations of gallidermin (200 and 400 μg/ml), concentrations that were substantially higher than the obtained MICs. Furthermore, the viability of fibroblasts was not influenced by gallidermin, and remained stable despite the high concentrations of gallidermin. This also means that gallidermin, as opposite to plantaricin NC8 αβ mentioned above, does not promote cell proliferation in fibroblasts. Cytotoxic effects can also be correlated to the release of LDH, an exclusive intracellular enzyme. Gallidermin‐treated cells at the two highest concentrations displayed significantly lower levels of LDH, and this together with the other tested concentrations, demonstrate that gallidermin has no cytotoxic effects on fibroblasts. Another study also analyzing the release of LDH from human gallidermin‐exposed peripheral blood mononuclear cells, showed however pronounced variations, making it hard to draw any conclusions regarding the efficacy of gallidermin (Kindrachuk et al., 2013).

Cell cytotoxicity was also assessed by the capacity of gallidermin to induce hemolysis of human RBCs, which is considered as a valid method for in vitro toxicity assessment. A degree of hemolysis at 0%–9% is considered as nontoxic (Pagano & Faggio, 2015). All tested concentrations of gallidermin, up to 400 μg/ml, caused less than 1% hemolysis. This is in accordance with a study that investigated the effect of gallidermin on sheep erythrocytes (Maher & McClean, 2006). However, cationic antimicrobial peptides can interact with other blood components (Yu et al., 2015). We therefore tested the ability of gallidermin to induce cell aggregation and release of ROS in whole blood and found that neither cell aggregation nor ROS production occurred.

We also investigated if gallidermin *per se* is able to induce inflammatory responses in dermal fibroblasts and analyzed the proinflammatory cytokines CXCL8 and IL‐6. We have previously shown that dermal fibroblasts release a wide array of inflammatory mediators and hence, this cell type is an important part of the innate inflammatory response (Palm, Khalaf, & Bengtsson, 2013). CXCL8 is a chemokine that recruits neutrophils to the site of infection, whereas IL‐6 is an important proinflammatory cytokine and an important mediator in acute inflammation. The highest tested concentration of gallidermin (400 μg/ml) significantly increased CXCL8 secretion compared to the control. Small amounts of IL‐6 were also significantly induced by 400 μg/ml of gallidermin. Lower concentrations of gallidermin did not elevate CXCL8 and IL‐6 above the unstimulated control. This indicates that gallidermin only has a minor capacity to raise an inflammatory response. Kindrachuk et al. investigated the innate immunity modulatory function of lantibiotics, including nisin and gallidermin, on human peripheral blood mononuclear cells, and found that nisin indeed was able to modulate host responses, including release of CXCL8, Gro‐α, and MCP‐1. However, gallidermin was more modest, which is in accordance to our study (Kindrachuk et al., 2013). Maher & McClean (2006) investigated the cytotoxic effects of gallidermin, nisin, and vancomycin on epithelial cells and found low cytotoxicity, especially regarding gallidermin. This supports our findings, which demonstrate that gallidermin is nontoxic to cells, does not affect cell viability and is not an inducer of inflammatory responses.

Knowledge of the pharmacodynamics of an antimicrobial agent, with rate and extent of bactericidal activity, bioavailability, elimination, and postantibiotic effect, provides a more rational basis for the determination of optimal dosing compared to MIC and MBC. Despite this, the antimicrobial activity of an antimicrobial agent is usually assessed by determining MIC and MBC in vitro. These conditions are probably rather different from those found at the actual site of infection and they may not reflect the in vivo effect. For example, the in vitro inoculum of bacteria, used for MIC and MBC, is in the exponential phase of growth, in contrast to an established infection in vivo where the majority of the bacteria have stopped dividing. MIC and MBC are also determined against a standard bacterial inoculum that does not necessarily correspond to bacterial densities at the site of infection (Levison & Levison, 2009). Therefore, it is important to elucidate these different aspects regarding gallidermin. In a first attempt, we studied the effects of gallidermin in an in vitro infection model. Primary human dermal fibroblasts were treated with *S. epidermidis* and *S. aureus*, respectively. As expected, *S. epidermidis* and *S. aureus* infected the cells, causing cell death and a considerable bacterial growth. Interestingly, these effects were more or less reversed by gallidermin. Fibroblast release of both CXCL8 and IL‐6 were markedly elevated by the various staphylococcal strains at both MOI:1 and MOI:10. The two *S. epidermidis* strains caused similar inflammatory responses, with RP62A inducing a slightly higher response. Although the strains displayed the same MIC values, gallidermin more effectively abolished *S. epidermidis* 117‐induced secretion of CXCL8 and IL‐6 from fibroblasts. This suggests that strain 117 is slightly more susceptible to gallidermin than RP62A, even though the two strains showed the same MIC value. Our finding that gallidermin totally abolishes bacteria‐induced release of CXCL8 and IL‐6 is very impressive and important, since it thereby may counteract harmful inflammatory processes.

It is reasonable to assume that pathogen‐associated molecular patterns expressed by the bacteria would induce a release of cytokines from fibroblasts even when the bacteria is killed by gallidermin. This was, however, not the case and this might be due to the fact that fibroblasts are not professional immune cells. On the other hand, this could also suggest that gallidermin has anti‐inflammatory effects.

Fibroblasts stimulated with MRSA released high amounts of both CXCL8 and IL‐6, an effect that was effectively antagonized by gallidermin. Interestingly, MRSA induced less of cytokines at MOI:10 compared to MOI:1, which may be due to a more pronounced cell death and subsequently a lower cytokine production and secretion. MSSA caused, compared to the other strains, a more modest secretion of the inflammatory mediators. In addition, gallidermin was less efficient in suppressing MSSA‐induced CXCL8 and IL‐6.

Overall, MRSA displayed a much higher gallidermin susceptibility compared to MSSA. MRSA differs from MSSA by modifications in the penicillin binding protein (PBP) 2A, making it methicillin‐resistant (Chambers, 1997; Hiramatsu, 1995; Hiramatsu, Cui, Kuroda, & Ito, 2001). If this modification of PBP2A also means that MRSA becomes more sensitive to gallidermin, or if other mechanisms lie behind this difference compared to MSSA, remains to be elucidated. An interesting study by Herbert et al., showed that *S. aureus* with a knockdown of the *dlt* operon, makes the mutant hypersensitive to various cationic antimicrobial peptides, including gallidermin. The *dlt* operon increases the positive charge of the cell wall, and hence, a *dlt* mutant becomes much more negatively charged due to lack of D‐alanine esters in the teichoic acids. The bacteria thereby become an attractive target for cationic antimicrobial peptides (Herbert et al., 2007). Overall, the bacteriocins complex mode of action means that it is rather difficult for pathogens to develop resistance, instead the bacteria rather gain a certain level of tolerance by increasing the number of positive charges in the cell envelope. Masking the cell envelope with positive charges gives unspecific protection against a broad variety of antimicrobials (Ernst & Peschel, 2011; Ernst et al., 2009; Gotz et al., 2014; Herbert et al., 2007; Peschel et al., 2001; Staubitz, Neumann, Schneider, Wiedemann, & Peschel, 2004). It is important to elucidate if bacteria can evolve resistance or tolerance against lantibiotics such as gallidermin. A previous study investigating the killing activity of gallidermin on biofilms, formed by *S. aureus* and *S. epidermidis*, revealed that a small subpopulation of 0.1%–1.0% of bacteria survived treatment, and these surviving bacteria need to be studied further (Saising et al., 2012).

Gallidermin shows great potential to treat infections caused by *S. epidermidis* and *S. aureus*, which both are clinically relevant species. *S. epidermidis* is a frequent colonizer of the skin and the mucous membranes, but rarely causes life‐threatening infections. Nevertheless, *S. epidermidis* is an opportunistic pathogen and the most common cause of nosocomial infections associated with indwelling medical devices due to its strong ability to attach to surfaces of foreign bodies and form biofilms. Hence, *S. epidermidis* is often associated with chronic infections and form biofilms that are very hard to eradicate, especially when multiresistant strains are involved. Antibiotic resistance is a widespread property of *S. epidermidis*, just like the case for the more virulent *S. aureus* (Otto, 2009). MRSA is a well‐known superbug that, together with other strains of *S. aureus*, are a major cause of hospital‐, livestock‐, and community‐acquired infections. This species is implicated in a wide array of infections, such as wound and skin infections, endocarditis, nosocomial pneumonia, osteomyelitis, and organ abscesses. Many of these infections can develop into lethal sepsis (Frieri, Kumar, & Boutin, 2017; Klevens et al., 2007; Stryjewski & Corey, 2014; Ventola, 2015). In fact, MRSA is causing thousands of deaths each year, and even though promising data show that MRSA infections is decreasing in some European countries and in USA, the mortality rate is still high, demanding new treatment strategies (Johnson, Hayes, Brown, Hoo, & Ethier, 2014; Kock et al., 2010; Purrello et al., 2016). A drawback of bacteriocins in clinical applications is the administration route. Small peptides such as gallidermin may be easily degraded. Therefore, it is important in future research to focus on peptide stability and how to improve it. For the present, we suggest that gallidermin could be a used as a topical agent against skin infections caused by *S. epidermidis* and *S. aureus*. Indeed, a paper demonstrated that a gel with gallidermin loaded in anionic niosomes delivered gallidermin into viable epidermis and dermis of rat skin (Manosroi et al., 2010).

This study demonstrates that gallidermin, besides effectively inhibiting *S*. *epidermidis* and *S*. *aureus*, especially MRSA, antagonizes bacteria‐induced inflammatory responses in dermal fibroblasts. We found that gallidermin shows no cytotoxic effects on fibroblasts, does not interact with blood components and has a low proinflammatory capacity. In summary, the encouraging bioactivities of gallidermin makes it an interesting candidate as a future antimicrobial substance for targeting infections caused by *S. aureus* and *S. epidermidis*.

## CONFLICT OF INTEREST

Authors have no competing financial interests.
